# Ohio State Meeting Jottings

**Published:** 1890-12

**Authors:** 


### Ohio State Meeting Jottings.

Dr. W. H. Sedgwick presided with dignity; his address is worth the close study of every one interested in dental education.

Dr. E. G. Betty, of Cincinnati, was elected president for '91. He will make a good one.

The DENTAL REGISTER for November was issued early so that copies could be sent to the meetings, all of which were eagerly sought for by those present.

Dr. J. R. Callahan has faithfully served the society as secretary since its reorganization. He was elected vice-president for '91, but in '92 will make a better president.

Dr. Otto Arnold succeeds to the secretaryship. He looks well since his European trip.

Dr. Frank Sage, of Cincinnati, is small in stature, but a giant in argument.

A number of names were voted upon for membership.

Dr. Jacobs, of Newark, gave a good plain talk about micoscopes, and how to use them.

Dr. Geo. H. Wilson, of Painesville, was elected second vice-president.

Dr. Fletcher, of Cincinnati, showed by his essay and illustrations, that his experiments were scientific, and productive of good to the profession. Read his paper carefully, then re-read it.

Dr. L. E. Custer, of Dayton, possesses good judgment and is an attentive listener.

The DENTAL REGISTER is *by* and *for* the dental profession.

Dr. C R. Butler is a deliberate and considerate speaker.

Drs. Dennis, of Portsmouth, and W. H. Todd, of Columbus, were among the healthy looking men at the meeting.

Prof. Detmer, of the Ohio State University is an enthusiast in micro-photography, and his demonstrations were highly entertaining and instructive.

The Senate Chamber is a fine place for a meeting, but a little too large for voices to be heard from a distance.

Dr. J. F. Callahan's paper on Hypnotism is a faithful exposition of the subject. It will be published in the next number.

Drs. C. M. Wright, T. S. Maxwell, Martha and J. E. Robinson, F. C. Runyan, and C. H. Harroun, all well-known members of the soci-ty, were missed from the meeting.

The dental instrument dealers made good displays.

All went home feeling benefited by the meeting.

Finis. READ THE DENTAL REGISTER. W.

